# Risk of Surgeon Contracting COVID-19 while Operating on COVID-19-Positive Patient, Impact of Safety Measures: Lessons Learnt

**DOI:** 10.1055/s-0042-1755619

**Published:** 2022-08-22

**Authors:** Mandar Koranne, Pratik D. Patil, Suchin S. Dhamnaskar

**Affiliations:** 1 Department of General Surgery, Seth Gordhandas Sunderdas Medical College and King Edward Memorial Hospital, Mumbai, Maharashtra, India

**Keywords:** COVID-19, surgeon, safety measure, operation room

## Abstract

**Introduction** On March 11, 2020, the novel coronavirus disease 2019 (COVID-19) was declared as a pandemic. General surgeons provide care to COVID-19 positive patients requiring emergency surgeries and hence are exposed to the virus. Surgery on COVID-19-positive patient itself is a major risk factor for surgeon to contract COVID-19 infection. Noticeably, there is no data regarding number of surgeons who have contracted COVID-19 after operating on COVID-19-positive patients. Hence, the aim of this study was to find out the exact incidence of COVID-19 among surgeons operating on COVID-19-positive patients and to analyze the impact of safety measures practiced by us.

**Methodology** The study was conducted in a tertiary care center in Mumbai. It was a retrospective observational study with duration of 5 months from May 1, 2020, to September 30, 2020. Only those surgeons (faculty and resident doctors) were included who performed surgeries on COVID-19-positive patients (diagnosed by reverse-transcription polymerase chain reaction [RT-PCR] test) and gave consent for participation. As an institutional protocol, all patients undergoing surgery were tested by RT-PCR test (irrespective of chest X-ray or symptoms). Nasopharyngeal swabs for COVID-19 disease were collected prior to procedure but in some of these, results came after surgery. Still such patients were included in this study. Irrespective of COVID-19 status, same precautions were taken for all surgeries. The details of the patients like date of surgery, age, sex, surgery performed, duration of surgery, type of anesthesia used, and operating surgeon were noted from operation room (OR) register. Details of surgeons (faculty and resident doctors) who fulfilled inclusion criteria were noted by interview in terms of their demographic parameters, such as age, sex, designation, experience in years after completing postgraduation, comorbidities, whether they ever contracted COVID-19 (if yes, date), and safety measures practiced (yes, no, or cannot recollect). Patient was assumed to be the source only if the surgeon contracted COVID-19 within 14 days of surgery.

**Results** A total of 34 surgeons (7 faculty and 27 residents) conducted 41 surgeries on COVID-19-positive patients during the study period. All of them gave consent for participation in the study. More than one surgeon was involved in a particular surgery. Hence, there were 78 occasions (faculty during 16 occasions and resident doctors on 62 occasions) when surgeons were at risk to contract COVID-19 while operating on patients ( *n* = 78). These surgeries had similar/comparable risk of COVID-19 exposure to surgeons and procedures with excessive exposure risk like airway procedures did not happen during the study period. The mean age of surgeon was 27.92 years ( *n* = 78, standard deviation = 5.71) and median experience of faculty after completion of postgraduate degree was 7 years ( *n* = 16, interquartile range [IQR] = 1.25–11.0). Only one faculty had comorbidity (diabetes mellitus). Duration of surgeries ranged from 50 to 420 minutes with median being 190 minutes ( *n* = 41, IQR = 120–240). Only one surgeon (male faculty) contracted COVID-19 within 14 days of surgery (1.3% incidence, *n* = 78), a total of seven surgeons contracted COVID-19 during study period but not within 14 days of surgery (source other than patient operated) and all remaining surgeons were asymptomatic throughout the study period. The surgeon who contracted COVID-19 (within 14 days) performed surgery for 260 minutes and under general anesthesia. All the surgeons followed standard steps of donning and doffing, used personal protective equipment (PPE) body cover, shoe cover, hood, double pair of gloves, and N-95 masks at all times ( *n* = 78). Intubation box was used in 100% cases of general anesthesia ( *n* = 19). Fogging of OR after each surgery and interval of 20 minutes between surgeries was followed in 100% cases. Also, patient was wearing mask at all possible times and anesthetist and support staff used PPE during all surgeries. Hence the relationship between COVID-19 status and these safety measures cannot be assessed. Goggles and face shields were not used on 88.5% ( *n* = 78) and 93.2% ( *n* = 73, because five surgeons could not recollect whether they used face shields or not) occasions, respectively. Also, immediate shower after surgery was not taken on 93.6% occasions ( *n* = 78). The surgeon who contracted COVID-19 had neither used goggles nor face shield. Also, he did not take shower immediately after surgery. However, there was no significant association between use of goggles, face shields, or shower after surgery and contraction of COVID-19 after operating patients (Fisher's exact *p* = 1.000). Air conditioner was switched-off only in 7.3% surgeries ( *n* = 41). Smoke evacuator (cautery with attached suction) was not used in 97.6% cases. Clinical documentation (handling of patient's files) was done outside OR in only 17.1% surgeries ( *n* = 41). However, there was no significant association between these safety measures and contraction of COVID-19 (Fisher's exact *p* = 1.000). General anesthesia was used in 19 surgeries (46.3%) while spinal anesthesia in 16 surgeries (39%), local anesthesia in 5 surgeries (12.2%), and total intravenous anesthesia (TIVA) in one surgery (2.4%). However, there was no significant association between type of anesthesia given during surgery and contraction of COVID-19 after operating on patients with Fisher's exact *p* -value of 1.000.

**Conclusion** Even though safety measures, like goggles, face shield, switching-off of air conditioner, use of smoke evacuator, and shower, immediately after surgery were not practiced in majority of cases, surgeon positivity rate was significantly less. Also, there was no use of negative pressure in OR. Hence, their significance becomes questionable. Although adopting all universal safety measures is in everyone's best interest, it is seldom cost-effective. To reduce resource exhaustion, especially in a pandemic situation, the use of various safety measures and staff must be balanced. Use and promotion of unnecessary safety measures leads to added health care costs and fear among health care workers in case of unavailability. Even though our study has a small sample size and has its own limitations, it can guide future studies to strengthen recommendations and reduce health care costs. This will also help in future epidemics/pandemics.

The World Health Organization (WHO) declared the novel coronavirus disease 2019 (COVID-19) as a public health emergency of international concern on January 30, 2020. 1 2 On March 11, 2020, COVID-19 was declared as a pandemic. 1 3 4 5 Health care workers who are in direct contact with patients are three times more likely to get admitted due to COVID-19 than health care workers who are indirectly involved in patient care. 6

General surgeons provide care to COVID-19-positive patients requiring emergency surgeries and hence are exposed to the virus. 7 Surgery on COVID-19-positive patient itself is a major risk factor for surgeon to contract COVID-19 infection. Noticeably, there are no data regarding number of surgeons who have contracted COVID-19 after operating on COVID-19-positive patients. We noticed this number to be surprisingly low at our department. Hence this study was planned to find out the exact incidence of COVID-19 among surgeons operating on COVID-19-positive patients and to analyze the impact of safety measures practiced by us.

## Objectives

The aim of this study was to find out the number of surgeons contracting COVID-19 after performing surgery on COVID-19-positive patients in a tertiary care center in Mumbai and to find out the impact of each safety measure undertaken while performing surgery.

## Methodology

The study was conducted in a tertiary care center in Mumbai after obtaining institutional ethics committee approval. It was a retrospective observational study with duration of 5 months from May 1, 2020, to September 30, 2020. Only those surgeons (faculty and resident doctors) were included who performed surgeries on COVID-19-positive patients (diagnosed by RT-PCR test) and gave consent for participation. As an institutional protocol, all patients undergoing surgery were tested by RT-PCR test (irrespective of chest X-ray or symptoms). Nasopharyngeal swabs for COVID-19 disease were collected prior to procedure but in some of these, results came after surgery. Still such patients were included in this study. Irrespective of COVID-19 status, same precautions were taken for all surgeries.

The details of the patients, like date of surgery, age, sex, surgery performed, duration of surgery, type of anesthesia used, and operating surgeon, were noted from operation room (OR) register. Details of surgeons (faculty and resident doctors) who fulfilled inclusion criteria were noted by interview in terms of their demographic parameters, such as age, sex, designation, experience in years after completing postgraduation, comorbidities, whether they ever contracted COVID-19 (if yes, date), and safety measures practiced (yes, no, or cannot recollect). Safety measures included the following: whether followed standard steps of donning personal protective equipment (PPE; including body cover, shoe cover, and hood), N95 mask, goggles, face shield, double pair of gloves, intubation box was used by anesthetist during induction of anesthesia (for general anesthesia cases), air conditioner was switched-off, smoke evacuator (cautery with attached suction) used, followed standard steps of doffing PPE, clinical documentation (handling of patient's file) done outside OR, shower taken immediately after surgery, fogging of OR after each surgery, 20-minute interval between two surgeries, patient wearing mask (at all possible times, i.e., preoperative, intraoperative, and postoperative), and anesthetist and support staff wearing PPE. Every surgeon was given the details of surgeries in which he/she was involved during the study period, for ease of remembrance.

Patient was assumed to be the source only if the surgeon contracted COVID-19 within 14 days of surgery (maximum incubation period is assumed to be 14 days according to WHO and Ministry of Health and Family Welfare [MOHFW], India guidelines). 8 9

## Results

A total of 34 surgeons (7 faculty and 27 resident doctors) conducted 41 surgeries on COVID-19-positive patients during the study period. All of them gave consent for participation in the study. More than one surgeon was involved in a particular surgery. Hence, there were 79 occasions when surgeons were at risk to contract COVID-19 while operating on patients. One occasion was omitted from the analysis because one faculty surgeon was COVID-19 positive and recovered before performing one particular surgery. Hence, he was likely to have intrinsic antibodies against the virus and we cannot assess effectiveness of the safety measures practiced. So, 78 occasions (faculty during 16 occasions and resident doctors on 62 occasions) were considered for statistical analysis ( *n* = 78).

These surgeries had similar/comparable risk of COVID-19 exposure to surgeons and procedures with excessive exposure risk like airway procedures did not happen during the study period.

The mean age of surgeon was 27.92 years ( *n* = 78, standard deviation [SD] = 5.71) and median experience of faculty after completion of postgraduate degree was 7 years ( *n* = 16, IQR = 1.25–11.0). Only one faculty had comorbidity (diabetes mellitus). Duration of surgeries ranged from 50 to 420 minutes with median being 190 minutes ( *n* = 41, IQR = 120–240). Only one surgeon (male faculty) contracted COVID-19 within 14 days of surgery (1.3% incidence, *n* = 78), a total of 7 surgeons contracted COVID-19 during study period but not within 14 days of surgery (source other than patient operated) and all remaining surgeons were asymptomatic throughout the study period. The surgeon who contracted COVID-19 (within 14 days) performed surgery for 260 minutes and under general anesthesia ( Table 1 ).

**Table 1:** Descriptive characteristics of surgeons who operated on COVID-19-positive patients during study period

Variables	Results	*n* (%)/mean (SD)/median (IQR)
Sex ( *n* = 78)	Male	50 (64.1)
	Female	28 (35.9)
Age in years ( *n* = 78)		27.92 (5.17)
Designation ( *n* = 78)	Faculty	16 (20.5)
	Resident	62 (79.5)
Faculty ( *n* = 16)	Assistant professor	15 (93.7)
	Professor	1 (6.3)
Resident doctor ( *n* = 62)	First year resident	2 (2.9)
	Second year resident	28 (45.2)
	Third year resident	30 (48.4)
	Senior resident	2 (3.2)
Experience in years ( *n* = 16)		7.0 (1.25, 11.0)
Comorbidities ( *n* = 78)	Yes	1 (1.3)
	No	77 (98.7)
Duration of surgeries in minutes ( *n* = 41)		190.0 (120.0, 240.0)
Number of surgeries one particular surgeon was scrubbed in ( *n* = 78)		3.0 (2.0, 4.0)

Abbreviations: COVID-19, novel coronavirus disease 2019; IQR, interquartile range; SD, standard deviation.

All the surgeons followed standard steps of donning and doffing, used PPE body cover, shoe cover, hood, double pair of gloves, and N-95 masks at all times ( *n* = 78). Intubation box was used in 100% cases of general anesthesia ( *n* = 19). Fogging of OR after each surgery and interval of 20 minutes between surgeries was followed in 100% cases. Also, patient was wearing mask at all possible times and anesthetist and support staff used PPE during all surgeries. Hence, the relationship between contraction of COVID-19 and these safety measures cannot be assessed.

Goggles and face shields were not used on 88.5% ( *n* = 78) and 93.2% ( *n* = 73, because five surgeons could not recollect whether they used face shields or not) occasions, respectively. Also, shower immediately after surgery was not taken on 93.6% occasions ( *n* = 78). The surgeon who contracted COVID-19 had neither used goggles nor face shield. Also, he did not take shower immediately after surgery. However, there was no significant association between use of goggles, face shields, or shower immediately after surgery and contraction of COVID-19 after operating patients (Fisher's exact *p* = 1.000).

**Fig. 1:**
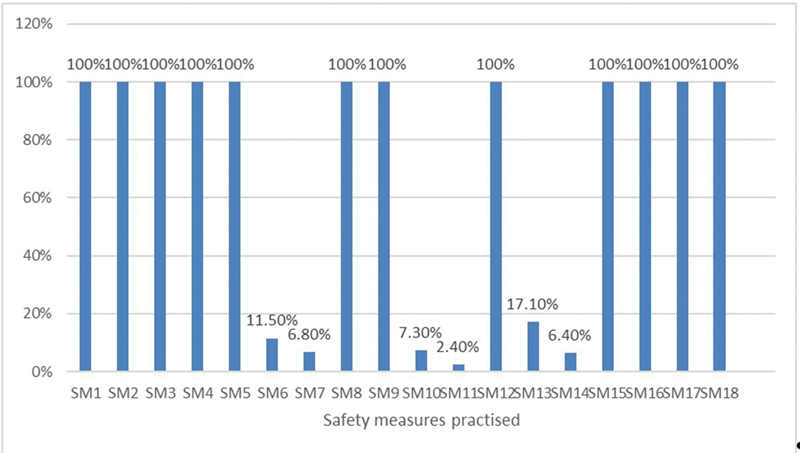
Frequency of use of individual safety measure. PPE, personal protective equipment; SM, safety measure; SM1, followed standard steps of donning PPE; SM2, used PPE body cover; SM3, used PPE shoe cover; SM4, used PPE hood; SM5, used N95 mask; SM6, used goggles; SM7, used face shield; SM8, used double pair of gloves; SM9, intubation box was used by anesthetist for induction in general anesthesia cases; SM10, air conditioner was switched-off; SM11, used smoke evacuator; SM12, followed standard steps of doffing PPE; SM13, clinical documentation of medical records done outside OR; SM14, shower taken immediately after surgery; SM15, fogging of OR after each surgery; SM16, 20-minute interval between two surgeries; SM17, patient wearing mask at all possible times; SM18, anesthetist and support staff wearing PPE.

Air conditioner was switched-off only in 7.3% surgeries ( *n* = 41). Smoke evacuator (cautery with attached suction) was not used in 97.6% cases. While, clinical documentation (handling of patient's files) was done outside OR in only 17.1% surgeries ( *n* = 41). However, there was no significant association between these safety measures and contraction of COVID-19 (Fisher's exact *p* = 1.000; Fig. 1 and Table 2 ). General anesthesia was used in 19 surgeries (46.3%) while spinal anesthesia in 16 surgeries (39%), local anesthesia in 5 surgeries (12.2%), and total intravenous anesthesia (TIVA) in one surgery (2.4%). However, there was no significant association between type of anesthesia given during surgery and contraction of COVID-19 after operating on patients with Fisher's exact *p* -value of 1.000.

**Table 2:** Statistics of safety measures where there is no significant association between safety measure and contraction of COVID-19 a

Safety measure	If contracted COVID-19 within 14 days of surgery		Total
SM 6 ( *n* = 78)	No ( *n* = 77)	Yes ( *n* = 1)	
Yes (%)	9 (11.7)	0 (0.0)	9 (11.5)
No (%)	68 (88.3)	1 (100.0)	69 (88.5)
SM 7 ( *n* = 73)	No ( *n* = 72)	Yes ( *n* = 1)	
Yes (%)	5 (6.9)	0 (0.0)	5 (6.8)
No (%)	67 (93.1)	1 (100.0)	68 (93.2)
SM 10 ( *n* = 41)	No ( *n* = 40)	Yes ( *n* = 1)	
Yes (%)	37 (92.5)	1 (100.0)	38 (92.7)
No (%)	3 (7.5)	0 (0.0)	3 (7.3)
SM 11 ( *n* = 41)	No ( *n* = 40)	Yes ( *n* = 1)	
Yes (%)	1 (2.5)	0 (0.0)	1 (2.4)
No (%)	39 (97.5)	1 (100.0)	40 (97.6)
SM 13 ( *n* = 41)	No ( *n* = 40)	Yes ( *n* = 1)	
Yes (%)	7 (17.5)	0 (0.0)	7 (17.1)
No (%)	33 (82.5)	1 (100.0)	34 (82.9)
SM 14 ( *n* = 78)	No ( *n* = 77)	Yes ( *n* = 1)	
Yes (%)	5 (6.5)	0 (0.0)	5 (6.4)
No (%)	72 (93.5)	1 (100.0)	73 (93.6)

Abbreviations: COVID-19, novel coronavirus disease 2019; SM6, used goggles; SM7, used face shield; SM10, air conditioner was switched-off; SM11, used smoke evacuator; SM13, clinical documentation of medical records done outside Operation Room; SM14, shower taken immediately after surgery.

a For other safety measures, we cannot assess the relationship between safety measure and contraction of COVID-19.

## Discussion

Through well-conducted research studies, it has become clear that adopting universal pandemic precautions is in everyone's best interest. 10 Various studies and guidelines have been published for safe surgical practices in COVID-19 pandemic. 11 12 13 14 15 But all the recommendations for safety measures are based on universal safety precautions and prior experiences related to management of surgical patients during previous epidemics like severe acute respiratory syndrome (SARS), Middle East respiratory syndrome (MERS), Ebola virus disease, and others. 16 For example, based on experience with other respiratory viruses, consistent use of PPE and masks is recommended for health care workers during treatment of COVID-19 patients. 17 18 19 20 But there have been no studies to validate the efficacy of these safety precautions per se for COVID-19.

During the study period, only emergency open surgeries were performed at our institute. Only one surgeon contracted COVID-19 possibly from the patient he operated. This too cannot be ascertained since there are innumerable ways of contracting COVID-19. Nevertheless, even if we assume the patient to be the source of COVID-19 for that surgeon, incidence was only 1.3%.

In their study, Park et al concluded that duration of contact with COVID-19-positive patient played a major role in the spread of virus. 21 There is a possibility of severe illness when exposed to a higher virus load. 22 Although there are no studies correlating duration of surgery and exposure to viral load, it can be stated that the longer the duration, the higher is the risk of contracting COVID-19 illness and with increased severity as well. In our study, the surgeon who contracted COVID-19 performed surgery for 260 minutes (median = 190 minutes).

The viral load in the peritoneal fluid is high as compared with respiratory fluid. 23 Viral RNA has also been demonstrated in blood and feces of COVID-19 patients. 24 25 All surgical procedures potentially provoke aerosolization of the virus and put surgeons at risk. Both laparoscopic and open surgical procedures result in vapor forming maneuvers and hence aerosolize the virus. Also, there is a possibility of splash of body fluids coming in contact with eyes, nose, mouth while operating. Use of either N95 or FFP2 masks by surgeons is recommended by the Center of Disease Control and Prevention 26 and Wang et al. 27 Chen et al demonstrated conjunctiva to be a potential route of transmission of coronavirus. 28 So routine use of eye protection is recommended to avoid exposure to virus while performing surgery. 27 29 Similarly, along with surgical caps, use of surgical hood is also recommended for the aerosol-generating procedures. 27 29 Continuous use of masks by patients at all possible times has also been recommended to reduce transmission. 30 Use of double pair of gloves has also been recommended while operating. 27 30

In our study, N95 mask was used in 100% cases. Hence the relationship between COVID-19 status and use of N95 mask cannot not be assessed. But surgeons did not use goggles in 88.5% cases and face shields in 93.2% cases. Possible reasons for noncompliance can be hampered vision due to accumulation of fog on goggles and face shield, improper fitting of goggles over spectacles, and others. This might be because majority of the general surgeries we dealt with did not pose great risk of splash of body fluids. But for cardiovascular or oral surgeries, they might have a significant impact. To improve compliance, various antifogging measures have been described like application of antiseptic liquid (cetrimide or sterillium) over plastic/glass surfaces. 31 32

In the review of literature conducted by Chirico et al, 33 there was not enough evidence to either support or refute the fact that air-conditioning systems favor the spread of SARS-coronavirus-2 (SARS-CoV-2). However, in previous coronavirus epidemics of SARS-CoV-1 and MERS-CoV, heating, ventilation, and air conditioning (HVAC) systems were suspected of facilitating the spread of these viruses. The guidelines released by various agencies, like elimination of any air recirculation within the ventilation system, use of HVAC system, switching-off of air conditioners, and others, are based on these studies. 34 However, switching-off of the air conditioner causes excessive sweating and discomfort while operating, especially in PPE, which ultimately hampers decision-making. In our study, air conditioner was switched-off in only 7.3% surgeries and we did not find any significant association between this safety measure and contraction of COVID-19.

A smoke evacuator (cautery with attached suction) has been recommended by Prakash et al 3 and Livingston 35 It is based on the fact that coronavirus is present in body fluids and use of electrocautery may aerosolize the virus. Coronavirus has not yet been demonstrated in surgical smoke, although there are case reports of surgeons contracting papillomavirus rarely when surgical smoke exposure was suspected to be the source. 36 However, in our study, cautery with attached suction was not used in 97.6% cases. Hence, it is imperative to conduct further studies before recommending this as a safety precaution.

It is advised that clinical documentation (handling of patients file) of medical records must be done outside the OR. 30 Also, shower immediately after surgery as a protective measure against COVID-19 has been recommended in literature. 30 But, in our study clinical documentation was done outside OR in only 17.1% surgeries and only 6.4% surgeons took shower immediately after surgery. Hence, it is recommended to conduct further studies before suggesting these as safety precautions.

Induction of general anesthesia is an aerosol generating procedure. Hence, whenever possible regional anesthesia is to be preferred which is associated with decreased risk to surgical staff as stated by Shanthanna and Uppal. 37 38 However, in our study, we did not find significant association between type of anesthesia given during surgery and contraction of COVID-19 after operating on patients.

A review by Al-Benna 39 and Braude and Femling 40 suggested that at least 12 air flow changes per hour are necessary to maintain required environment. Air exchanges prevent air (and hence the virus) from stagnating in a particular area. Also, creation of negative pressure ORs with separate ventilation system is recommended. 11 27 A negative pressure room works on the principle of lower air pressure inside the room as compared with its surroundings. This prevents potentially harmful particles within the room to escape outside. Hence, people outside the room are protected from exposure. This should theoretically increase the exposure of people inside the room to the contaminant if it is not associated with air exchanges and use of high efficiency particulate air (HEPA) filter. HEPA filter fitted in air handling unit (AHU) that filters out viruses, and thereby reduces the viral load of environment both inside and outside of the OR. Our center does not have negative pressure OR. Hence, larger studies are required to support or refute their role in surgeon's protection against COVID-19. The cost of constructing negative pressure ORs can be reduced which will significantly reduce health care costs especially in low-income countries.

Coccolini et al suggested that patients requiring surgery must be treated as COVID-19-positive until proven otherwise to minimize the chances of infection. 30 In emergency situations, it is not feasible to wait for swab report and life-saving surgeries have to be performed as recommended by systematic review done by De Simone et al 41 and study conducted by Gök et al. 42 This mandates use of safety measures in all patients requiring emergency surgery 41 and adds to health care costs. Hence it is necessary to conduct larger studies to evaluate the need of each safety measures. This will help to reduce financial burden on health care system by decreasing the number of unnecessary safety measures.

## Limitations

This study is retrospective observational study. No COVID-19 RT-PCR testing of operating surgeon was done until symptomatic as per institutional guidelines. Hence, asymptomatic carrier is the limitation of the study. Also, this data are limited to emergency open surgeries as elective and laparoscopic surgeries were not performed at our institute during study period.

## Conclusion

Even though safety measures, like goggles, face shield, switching-off the air conditioner, use of smoke evacuator, and shower immediately after surgery, were not practiced in majority of cases, surgeon positivity rate was significantly less. Also, there was no use of negative pressure OR. Hence, their significance becomes questionable. Although adopting all universal safety measures is in everyone's best interest, it is seldom cost-effective. Use and promotion of unnecessary safety measures lead to added health care costs and fear among health care workers in case of unavailability. Even though our study has a small sample size and has its own limitations, it can guide future studies to strengthen recommendations and reduce health care costs. This will also help in future epidemics/pandemics.
